# Supplementary material for: Measurement of charges and chemical bonding in a cryo-EM structure
Source: Commun Chem. 2023 May 31;6:98. doi: 10.1038/s42004-023-00900-x (PMC10232537; doi:10.1038/s42004-023-00900-x)
Supplement: Supplementary file 2 — Supplementary Information [file 42004_2023_900_MOESM2_ESM.pdf]

Supplementary Information

Measurement of charges and chemical bonding in a cryo-EM structure

Saori Maki-Yonekura, Keisuke Kawakami, Kiyofumi Takaba, Tasuku Hamaguchi, and Koji Yonekura

To whom correspondence should be addressed.

E-mail: yone@spring8.or.jp

Supplementary Discussion

1. Minimization of beam tilts off the axial coma-free axis

Large fluctuations in beam tilt occurred in our previous data collections using a CFE gun equipped in a CRYO ARM 300 microscope (JEOL), even though such images were collected exclusively through the use of mechanical stage shifts [1 - 5]. The data gave reconstructions to ~1.5 Å resolution [1,3]. Beam tilts from the axial coma-free axis caused a marked increase in phase errors in proportion to the third power of spatial frequencies. Although it is possible to estimate the beam tilts in acquired images and correct errors [6 - 8], such a post-correction scheme has its limitations particularly for larger beam-tilt errors at higher resolutions (see below).

The fluctuations sometimes range over several mrad [1-5], and this is likely due to off-pivot points of the beam on the specimen plane. Adjustment of the pivot points needs accurate compensation for beam tilts and shifts by means of condenser lens deflector coils. We had used the diffraction mode for compensation of beam shifts, as per instructions in the manual provided by JEOL. Beam tilt fluctuations still appeared significant despite simply changing the magnification without applying any image shifts. The fluctuations became much worse during data acquisition in a high throughput scheme where both stage and image shifts were implemented and one image stack per hole and 3 × 3 stacks from holes clustered around a central hole were taken once the stage moved to a new position (Supplementary Figs. 2a and c). This happened even though we had actively adjusted the beam tilt to the axial coma-free axis during data collection based on pre-calibration by SerialEM [9]. Post-estimation and correction of beam tilts for all image stacks separately provided Dataset B, which gave a 3D reconstruction to ~1.49 Å resolution by the gold standard FSC criteria (Supplementary Figs. 3a and d) [10].

We then adjusted compensation of beam shifts in imaging mode as done for the data acquisition condition (see also Methods and Efremov & Stroobants, 2021 [11]). SerialEM and JAFIS Tool (see Methods) were used to take image stacks over 5 × 5 holes per stage shift. This remarkably improved beam tilts in individual images comprising this dataset, named Dataset A, and most deviations were within ~ 0.15 mrad (Supplementary Figs. 2a and b), even though there were larger image shifts during image acquisition than in Dataset B. The obvious improvements provided by the new alignment method suggest that the effective sample height changes between diffraction and imaging modes. Thus, for the CRYO ARM microscope, it is important to compensate for the beam shifts and acquire data under the same condition.

Averaging and symmetrization are shown to reduce phase errors caused by beam tilts off the axial coma-free axis to some extent even without post-correction of phase shift by beam tilts [12, 13]. The post-correction can further improve the resolution [6]. The Rosenthal-Henderson plots [14] for Datasets A and B appear similar up to ~ 1.7 Å (Supplementary Fig. 3b), with the most prominent difference being the beam tilt fluctuations (Supplementary Fig. 2). Thus, the present data would suggest that the post-correction is probably effective to this resolution range but beyond this range becomes less so with such large beam tilt fluctuations.

The catalogue value of the spherical aberration coefficient, *Cs* = 2.7 mm, means that even 0.1 mrad off-estimation of beam tilt introduces significant phase errors in high-resolution ranges (Supplementary Fig. 2d). Errors are much worse with an accelerating voltage of 200 kV compared with 300 kV, and a new *Cs* corrector was introduced to remove phase errors by both on-axial and off-axial coma [15]. Nevertheless, our data indicate that the resolution can be

extended to sub-1.2 Å without this device. The *Cs* corrector and the monochromator would be impractical for many users due to instability, additional complexities in electron optics and operation, and high price.

2. Estimation of the standard uncertainties in bond lengths

In macromolecular X-ray crystallography, diffraction-component precision index (DPI) [16] is a standard metric for estimation of coordinate errors in the atomic model. According to Cruickshank, the root mean square differences (RMSD) in atomic positions $<\Delta r>$ between two structures solved independently are in good agreement with the estimated standard uncertainties of positions $\sigma(r)$ [16, 17]. For unrestrained refinement of crystal structures of small molecules, the standard uncertainty of a bond length between two atoms with similar coordinate errors is approximated by multiplying the coordinate error, i.e. DPI or $\sigma(r)$, by the square root of 2 [16, 17].

DPI relies on accuracy of diffraction intensity, but again CTF changes amplitude in EM images, yielding less accurate measures for amplitude. Still, REFMAC5 [18] can provide DPI during refinement of a model against a single-particle reconstruction, but we think a more suitable criterion is needed here. We calculated RMSD between two models obtained by restrained MD refinement against two half maps reconstructed independently (see Methods) to estimate the standard uncertainty in atomic positions in the model. The half maps are shown to contain information corresponding to F_{obs} and $\sigma(F_{obs})$ of X-ray crystallography [19 - 21]. We then plotted calculated values of DPI for various X-ray structures and those of $RMSD_{1/2}$ named here for cryo-EM reconstructions in a given resolution range (Supplementary Fig. 8; Supplementary Table 2). The graphs show consistency between them. Thus, $RMSD_{1/2}$ could be

used for estimation of coordinate errors in single-particle cryo-EM models. $RMSD_{1/2}$ for the apoferritin reconstruction in this study yields ~ 0.05 Å and this becomes ~ 0.05 × $\sqrt{2}$ = ~ 0.07 Å for the standard uncertainty between two atoms. Considering that $RMSD_{1/2}$ is calculated based on half maps and might be underestimated, differences between the average lengths of N-H and C-H_n bonds are 1 – 2 σ level (Table 1).

Supplementary Table 1: Data and refinement statistics.

	Dataset A	Dataset B
PDB code	8J5A	-
EMD code	EMD-35984	EMD-35981
EMPIAR code	EMPIAR-11534	EMPIAR-11532
**Data collection**		
EM Grid	Quantifoil R1.2/1.3	
Microscope	CRYO ARM 300	
Imaging device	K3	
Acceleration voltage (kV)	300	
Beam-tilt compensation	Imaging mode (× 100,000)	
Beam-shift compensation	Imaging mode (× 100,000)	Diffraction mode
Data collection software	SerialEM + JAFIS Tool	SerialEM
Coma-free alignment	JAFIS Tool	SerialEM
Image shifts per stage movement	5 × 5 holes (JAFIS Tool)	3 × 3 holes (SerialEM)
Imaging mode	CDS Super-resolution	Counted Super-resolution
Nominal magnification	× 100,000	
Total exposure time (sec)	2.34	0.8
No. of frames	40	50
Total electron exposure (e^- Å^{-2})	36.47	51.82
Defocus range (μm)	-0.5 - -1.0	-0.5 - -1.5
Physical pixel size (Å)	0.495	0.495
No. of total image sets	12,114	2,173
**Data processing**		
No. of used image sets	7,852	1,122
Final particles (no.)	2,235,864	311,583
Pixel size for final map	0.396	0.495
Symmetry imposed	*O*	*O*
Map resolution (Å)	1.19	1.49
FSC threshold	0.143	0.143
**Refinement**		
Initial model (PDB code)	7KOD	-

Model resolution (Å)	1.2	-
FSC threshold	0.5	-
Q-score for protein *	0.91	-
Estimated resolution (Å) by Q-score for protein *	1.18	-
Model composition in the asymmetric unit		
Non-hydrogen atoms	2248	-
Protein residues	172	-
Water	142	-
Ion	1 (Na^+)	-
B-factors ($Å^2$)		
Protein	19.5	-
Water	37.7	-
Ion	27.7	-
R.m.s. deviations		-
Bond lengths (Å)	0.013	-
Bond angles (Å)	1.55	-
Validation		
MolProbity score †	1.09	-
Clashscore	3.00	-
Poor rotamers (%)	0.75	-
Ramachandran plot		
Favored (%)	99.41	-
Allowed (%)	0.59	-
Disallowed (%)	0.00	-
$RMSD_{1/2}$ (Å) ‡	0.05	-

* An index showing resolvability of atoms, amino acid residues, and ligands assigned in a cryo-EM map [22].

† Defined in Williams et al. (2018) [23].

‡ Defined in this study.

Supplementary Table 2: Raw data for plots of DPI and $RMSD_{1/2}$.

Single-particle cryo-EM

PDB ID	Resolution	$RMSD_{1/2}$ *
8J5A †	1.19	0.05
7A4M	1.22	0.048
7KFR	1.56	0.095
7A5V	1.7	0.125
7VD8	1.96	0.156
7D1T	1.95	0.156
7KOD	1.66	0.058
7COY	2.58	0.3
7DD9	2.4	0.157
7P0Z	2.43	0.229

* Defined in this study.
† This study.

X-ray crystallography

PDB ID	Resolution	DPI	PDB ID	Resolution	DPI	PDB ID	Resolution	DPI
1IUA	0.80	0.008	6V7G	1.4	0.052	6Y2K	1.9	0.098
1MC2	0.85	0.012	3ASL	1.41	0.076	6XD2	1.9	0.117
1ALZ	0.86	0.015	7TM5	1.41	0.049	6V9K	1.9	0.129
1DY5	0.87	0.011	1B0B	1.43	0.042	16GS	1.9	0.187
1AB1	0.89	0.018	7R66	1.44	0.055	7RZP	1.95	0.145
1G66	0.90	0.015	2ADO	1.45	0.076	7KG5	1.95	0.145
1B0Y	0.93	0.022	2AGW	1.45	0.060	7JI2	1.95	0.131
1EA7	0.93	0.014	2AS6	1.45	0.056	7DZZ	1.95	0.141
1GVU	0.94	0.019	7S3U	1.45	0.057	6XL1	1.95	0.125
1HJ9	0.95	0.014	6YQG	1.45	0.04	6W50	1.95	0.098
1KTH	0.95	0.020	3AK3	1.48	0.099	6WI2	1.95	0.176
1LUG	0.95	0.016	7VB3	1.48	0.062	6PKU	1.95	0.115
1NKI	0.95	0.02	6Q7I	1.48	0.063	6PP0	1.97	0.181
1AHO	0.96	0.023	1ABS	1.5	0.075	7F2U	1.98	0.209
1K5C	0.96	0.016	1AKI	1.5	0.094	6PJB	1.98	0.205
1LUQ	0.96	0.018	1B13	1.5	0.074	101M	2.07	0.139
1BYI	0.97	0.016	1BIO	1.5	0.084	117E	2.15	0.249
1C75	0.97	0.015	1BW9	1.5	0.075	12GS	2.1	0.248
1F94	0.97	0.018	2AJU	1.5	0.096	1A0U	2.14	0.209
1GVT	0.98	0.017	2AX2	1.5	0.084	1A2U	2	0.197
1IXH	0.98	0.017	3ARV	1.5	0.056	1A4Y	2	0.2
1K4I	0.98	0.023	6STO	1.5	0.063	1A6R	2.05	0.181
1MNZ	0.99	0.016	6Q62	1.5	0.107	1A95	2	0.203
6YF3	1	0.027	6Q92	1.5	0.056	1A9R	2	0.262
6YDJ	1.04	0.024	2AVK	1.53	0.094	1AA4	2.1	0.224
6YQN	1.05	0.026	3AP7	1.53	0.071	2AX1	2.1	0.249
7LKL	1.05	0.026	1A7W	1.55	0.08	2B4I	2	0.198
6ZEG	1.09	0.021	1AVM	1.55	0.102	2BCX	2	0.226
1AKG	1.10	0.036	2A6X	1.55	0.094	2BRH	2.1	0.195
1BKR	1.10	0.029	6SSE	1.56	0.065	2C1F	2.1	0.174
1BQ8	1.10	0.025	6QBC	1.56	0.078	2D5V	2	0.203

5XTL	1.10	0.025	1A3H	1.57	0.07	2DJG	2.05	0.170
7LOB	1.1	0.031	2AEX	1.58	0.072	2DSA	2.1	0.208
7CIO	1.1	0.032	3AF7	1.58	0.071	2E2T	2.05	0.17
7CTS	1.1	0.025	3AJQ	1.58	0.070	7RYA	2.1	0.173
6YIY	1.11	0.027	6YPN	1.58	0.107	7PV8	2.05	0.263
6YF1	1.12	0.036	1G8S	1.6	0.059	7RT4	2.1	0.203
6YH7	1.12	0.031	1GI5	1.6	0.111	7NL7	2.1	0.204
1D2U	1.15	0.035	1GPQ	1.6	0.097	7K81	2	0.172
1H2J	1.15	0.026	1GY7	1.6	0.098	7GSP	2	0.191
1HDO	1.15	0.024	1J3Z	1.6	0.083	7E19	2.15	0.192
1J0O	1.15	0.028	368D	1.6	0.107	7E99	2.1	0.195
6YOL	1.15	0.032	7WKR	1.6	0.082	7FD0	2	0.179
7M10	1.15	0.032	7Q47	1.6	0.09	7D3I	2	0.208
7D8F	1.15	0.026	6ZCN	1.6	0.096	7D6W	2.15	0.204
1H4X	1.16	0.029	6YLM	1.6	0.082	7DBG	2.06	0.185
1HBN	1.16	0.030	6X8Q	1.6	0.089	7DY8	2.1	0.226
7C7D	1.16	0.030	6V8R	1.6	0.106	7BRO	2	0.189
1CZP	1.17	0.032	5XRN	1.6	0.075	7C9P	2	0.146
1H97	1.17	0.044	5WDR	1.6	0.115	7AO5	2.06	0.17
1HVB	1.17	0.026	5YJ7	1.61	0.139	7AWG	2	0.246
7DNC	1.17	0.039	7M2F	1.63	0.117	7AX7	2.05	0.197
7L3Y	1.18	0.027	1GW1	1.65	0.098	7BAG	2	0.221
1GU2	1.19	0.031	1J9Q	1.65	0.113	6ZGS	2.15	0.204
6Z5W	1.19	0.038	6ZGM	1.65	0.097	6Y6O	2.04	0.165
6YZH	1.19	0.038	7O4Z	1.67	0.103	6YDM	2.1	0.220
7LX9	1.19	0.039	6YPO	1.67	0.096	6X6Q	2.17	0.192
1AMM	1.2	0.05	6X6B	1.67	0.098	6XJK	2.02	0.211
1ATG	1.2	0.035	5ZOU	1.68	0.087	6XSE	2.1	0.267
1C0P	1.2	0.024	7MWV	1.69	0.107	6XXY	2.09	0.218
1CC7	1.2	0.043	1GC0	1.7	0.129	6UQD	2.02	0.204
2BK9	1.2	0.036	1GU7	1.7	0.081	6UUI	2.07	0.145
2C1V	1.2	0.04	1JSR	1.7	0.117	6UWD	2.04	0.166
2DLB	1.2	0.035	1JYE	1.7	0.1	6UY3	2	0.16
2DUH	1.2	0.046	6YZ4	1.7	0.129	6V29	2	0.158
2EU7	1.2	0.053	6W45	1.7	0.079	6VIM	2	0.123
3AKP	1.2	0.043	6WCG	1.7	0.11	5Z1O	2	0.209
3AUH	1.2	0.046	6VBS	1.7	0.068	5Z5F	2.1	0.198
3B12	1.2	0.039	5VZN	1.7	0.135	5YH8	2.12	0.212
3BQP	1.2	0.054	1G81	1.71	0.139	5YL6	2	0.161
3BVW	1.2	0.032	6VH9	1.71	0.165	5XWL	2.1	0.227
7REK	1.2	0.046	7NNQ	1.73	0.105	5XZ9	2	0.194
7P8B	1.2	0.045	1G60	1.74	0.106	5XA2	2.03	0.196
2ARM	1.23	0.045	1JYV	1.75	0.104	5XDU	2	0.213
3C5A	1.23	0.050	5ZHJ	1.75	0.119	5XIE	2.05	0.142
2BJI	1.24	0.062	5ZPS	1.75	0.103	5XMX	2	0.195
2DEA	1.24	0.042	5ZYC	1.75	0.092	5XVG	2.1	0.234
3AWS	1.24	0.041	5Y12	1.75	0.07	7WEW	2.3	0.233
1C1D	1.25	0.043	7NA9	1.76	0.18	7RLZ	2.27	0.224
2B82	1.25	0.053	7LT8	1.76	0.119	7SEM	2.2	0.185
2BVR	1.25	0.053	6Z94	1.76	0.115	7T9W	2.2	0.25
2C60	1.25	0.045	6VR2	1.77	0.073	7VSO	2.35	0.237

2COV	1.25	0.042	5ZF9	1.77	0.077	7OOE	2.37	0.233
2DN1	1.25	0.06	7OC0	1.78	0.125	7P3G	2.39	0.249
3AYI	1.25	0.029	1GSX	1.79	0.152	7RCA	2.26	0.243
3B0G	1.25	0.036	7S6F	1.8	0.115	7MZK	2.25	0.223
7QU5	1.25	0.038	7SKB	1.8	0.188	7NHE	2.23	0.176
7TM6	1.26	0.036	7V3B	1.8	0.182	7NMX	2.3	0.27
2CAK	1.27	0.022	7KMV	1.8	0.126	7OA5	2.38	0.27
2CC6	1.27	0.038	7KQQ	1.8	0.107	7LPQ	2.32	0.233
1C52	1.28	0.049	7L7C	1.8	0.165	7LVI	2.2	0.209
7PB5	1.28	0.034	7KC3	1.8	0.09	7MJV	2.24	0.262
1BK0	1.3	0.048	7EVR	1.8	0.1	7LG3	2.3	0.23
1BXA	1.3	0.053	7FH3	1.8	0.107	7KD3	2.3	0.234
2AQP	1.3	0.043	6YP3	1.80	0.113	6YMZ	2.3	0.188
2BIG	1.3	0.035	6XVH	1.80	0.149	6YTA	2.3	0.274
2BJS	1.3	0.039	6WZK	1.8	0.108	6XPT	2.3	0.279
3AK9	1.3	0.052	6X3R	1.8	0.113	6XSP	2.3	0.284
7S7Y	1.3	0.054	6XB1	1.8	0.122	6XZV	2.3	0.190
2BLO	1.33	0.044	6VW9	1.8	0.158	6Y6C	2.26	0.152
1BQK	1.35	0.061	6V94	1.8	0.091	6YG4	2.3	0.228
1BSM	1.35	0.08	6PFQ	1.8	0.078	6YG4	2.3	0.228
3B28	1.35	0.047	7JZA	1.82	0.072	6WRZ	2.25	0.292
3BOM	1.35	0.066	7L2A	1.85	0.175	6VUE	2.28	0.213
3BUB	1.38	0.058	7JST	1.85	0.186	6WHL	2.3	0.199
194L	1.4	0.069	6YOB	1.85	0.156	6UNR	2.2	0.232
1AWD	1.4	0.044	6W5J	1.85	0.156	6US5	2.25	0.299
1B16	1.4	0.068	5B66	1.85	0.108	6V55	2.38	0.296
1BVD	1.4	0.079	6YKD	1.86	0.094	6VK4	2.35	0.21
2ACF	1.4	0.051	5B5E	1.87	0.119	6TQJ	2.3	0.286
2AMS	1.4	0.066	7DIU	1.88	0.091	6TZV	2.39	0.232
2ANY	1.4	0.061	7PMU	1.89	0.135	6U4X	2.25	0.236
3ACH	1.4	0.061	9GAC	1.9	0.202	6UC6	2.32	0.213
3AGT	1.4	0.082	7KJ8	1.9	0.097	6UMS	2.34	0.241
3AI0	1.4	0.060	7K1C	1.9	0.139	6TFL	2.4	0.228
3AMC	1.4	0.066	7K99	1.9	0.183			
6YQ2	1.4	0.051	6XHP	1.90	0.144			

Supplementary Fig. 1: Fourier transform of a metal image.

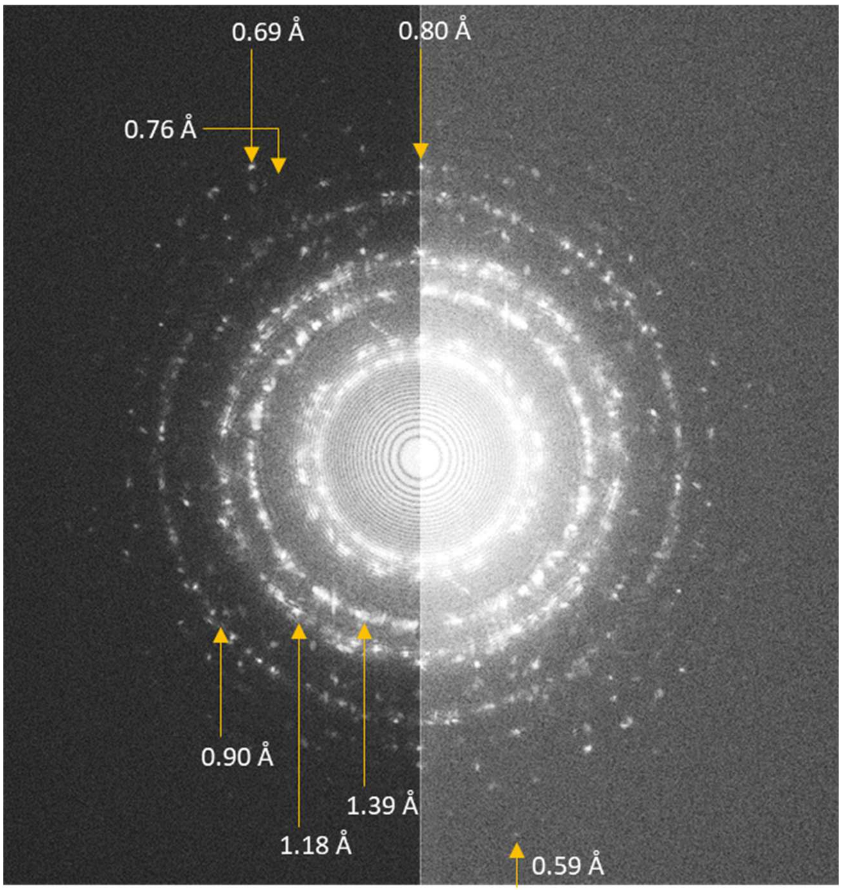

Carbon film deposited with Pt-Ir was imaged with a CFE gun at a nominal magnification of × 300,000 and defocus of ~ -1 µm. Beam illumination was focused and marginally off the parallel condition in order to give significant counts in each frame. Movie images were recorded on a K3 detector in counted mode at a dose rate of ~ 7 e^- $pixel^{-1}$ s^{-1} for 15 s exposure, and motion-corrected full frames were summed with DigitalMicrograph (Gatan, AMETEK). Signals to ~ 0.6 Å are visible. The right half is brightened for clarity of high-resolution signals.

Supplementary Fig. 2: Beam tilts from the axial coma-free axis.

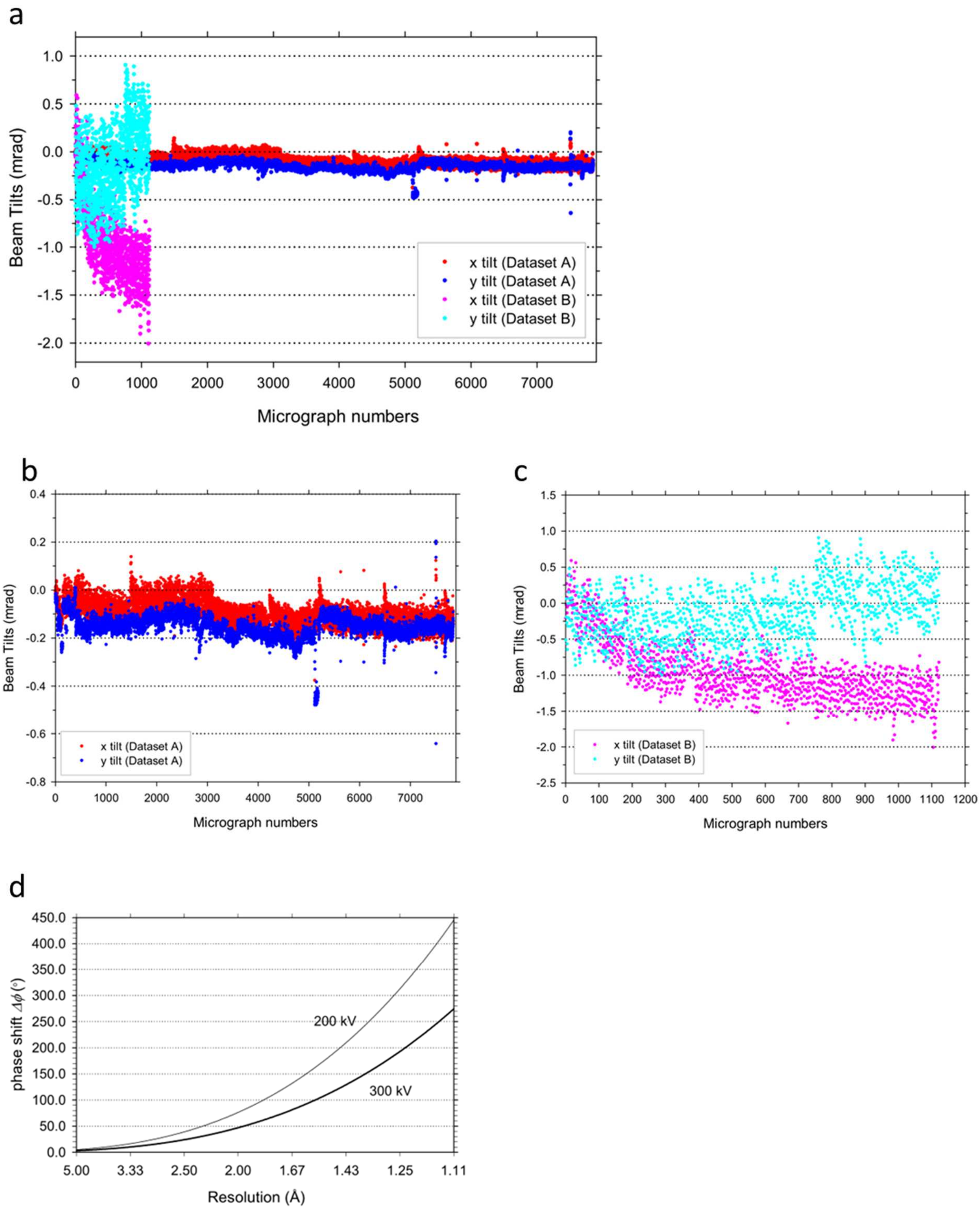

a Beam tilt variations during data collection. Compensation of beam shifts was pre-adjusted in imaging mode (Dataset A) and in diffraction mode (Dataset B). **b** Zoom-up of the plots for

Dataset A. **c** Zoom-up of the plots for Dataset B. **d** Plots of phase shift

$$\Delta\phi(\mathbf{k}) = 2\pi Cs\lambda^2 |\mathbf{k}|^3 b$$

introduced by an angle off the axial coma-free axis b = 0.1 mrad at a spatial frequency **k** with a spherical aberration coefficient Cs = 2.7 mm and the wavelength of electron λ = 2.51 pm for 200 and λ = 1.97 pm for 300 kV. The phase shifts become 112º at 1.5 Å resolution and 218º at 1.2 Å for 300 kV and a beam tilt of 0.1 mrad.

Supplementary Fig. 3: Comparison of Datasets A and B.

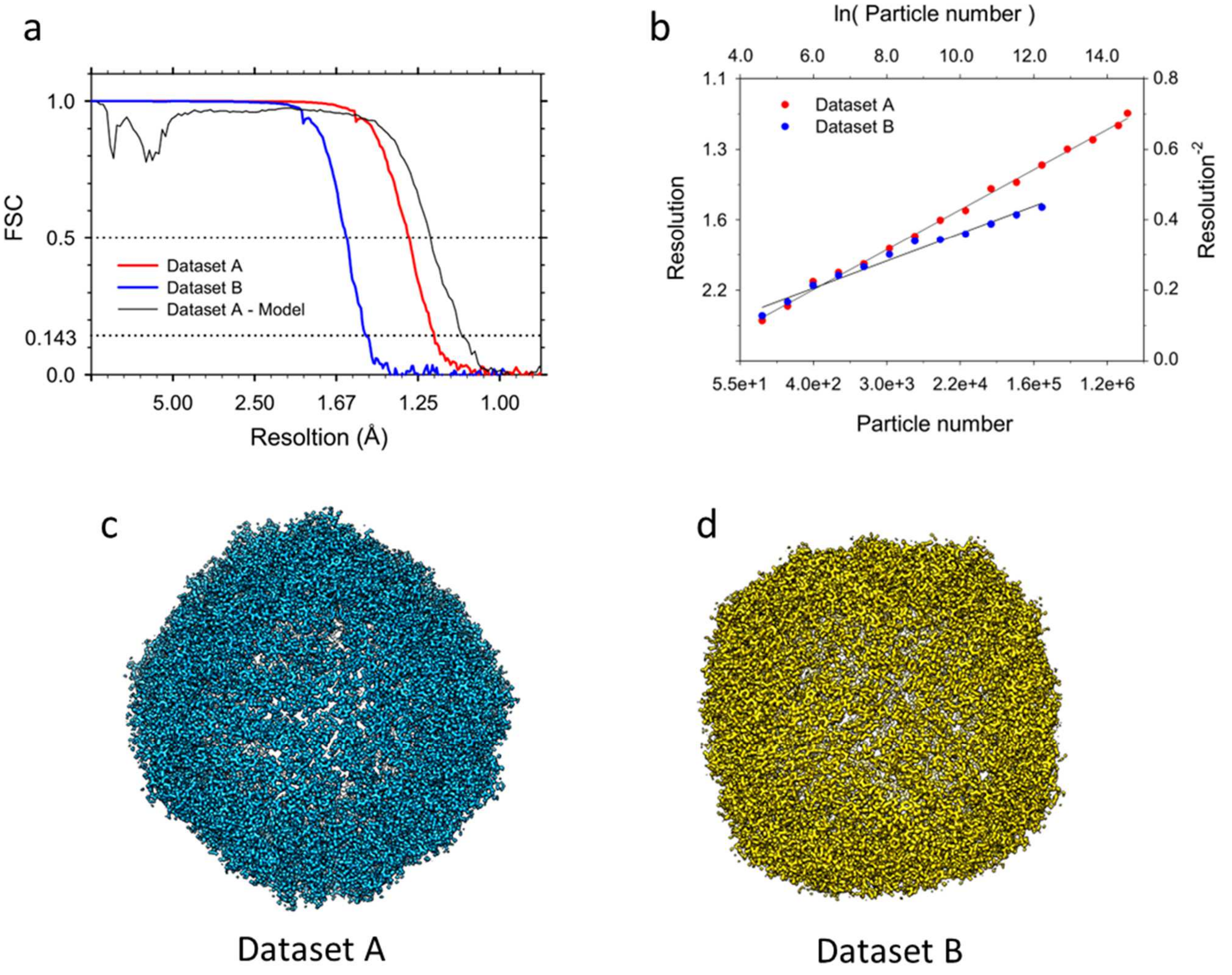

a FSC plots for half maps of Datasets A, B and Dataset A vs the model. **b** Rosenthal-Henderson

plots for Datasets A and B. The Rosenthal-Henderson B-factors are estimated as 34 Å^2 for Dataset A and 51 Å^2 for Dataset B. Reconstructions were repeated four times from subgroups consisting of ≤ 1600 randomly-selected particles, and Ewald-sphere correction was applied to subgroups of ≥ 6400 particles. **c** Reconstruction of Dataset A. **d** Reconstruction of Dataset B.

Supplementary Fig. 4: Other examples of structure details.

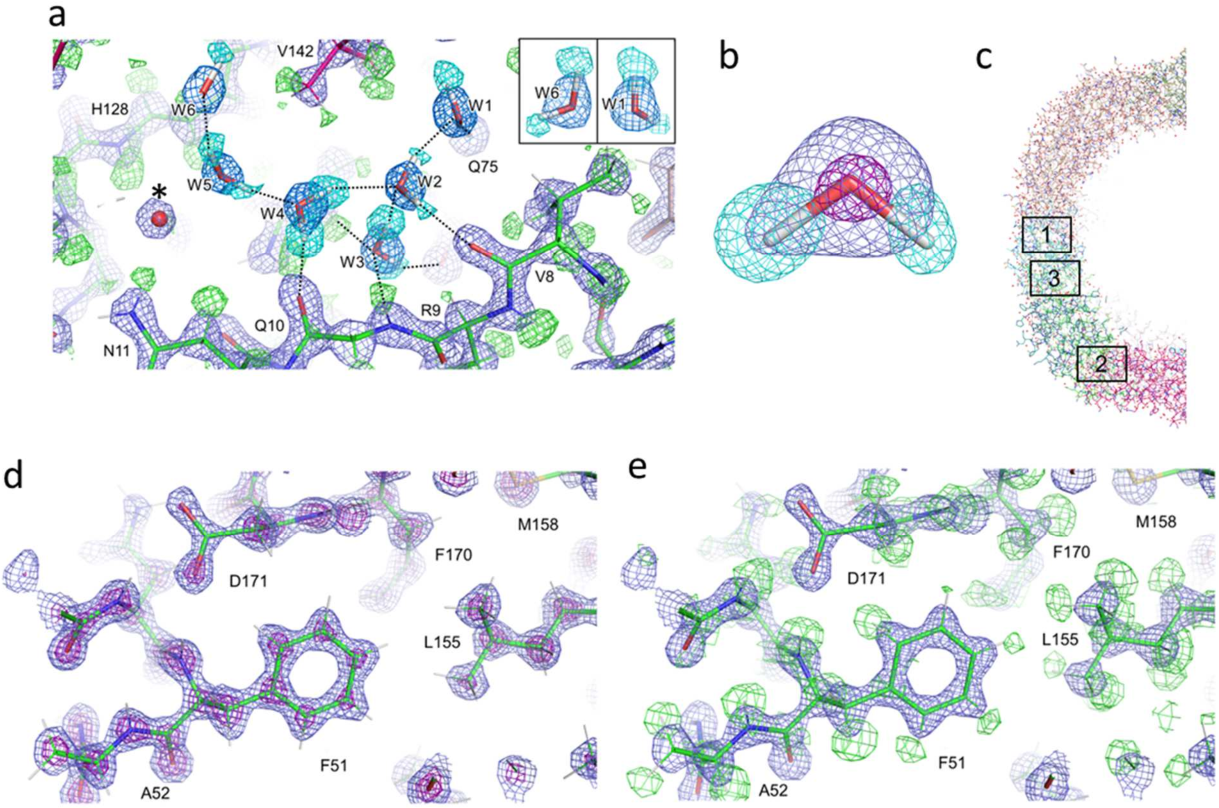

a View around a water cluster. Sky blue nets represent six water molecules (W1 – W6) showing two hydrogen densities in cyan and comprising a water cluster through hydrogen bonds. A water marked with '*' has no clear hydrogen densities. **b** One of the water molecules (W4) in a. **c** A cutout view of half the apoferritin complex indicating the area marked "1" for Figs. 1a and b, "2" for a and "3" for d and e. **d**, **e** View around a phenylalanine (Phe 51). Displayed as

in Figs. 1a and b except for c. The density levels of blue in a, b and d – e and purple nets in b and d are 2σ and 7σ, respectively. Sky blue nets for water in a is at 2σ. Green nets in a, e and cyan nets in a and b are at 2.5 - 3σ. Cyan nets in insets of a are at 2σ for clarity of W1 and W6. W1, W2, W3, W4, W5, W6, and '*' are numbered 348, 391, 335, 375, 385, 333, and 310, respectively, in the PDB.

Supplementary Fig. 5: Amino-acid residues exhibiting multi-conformations.

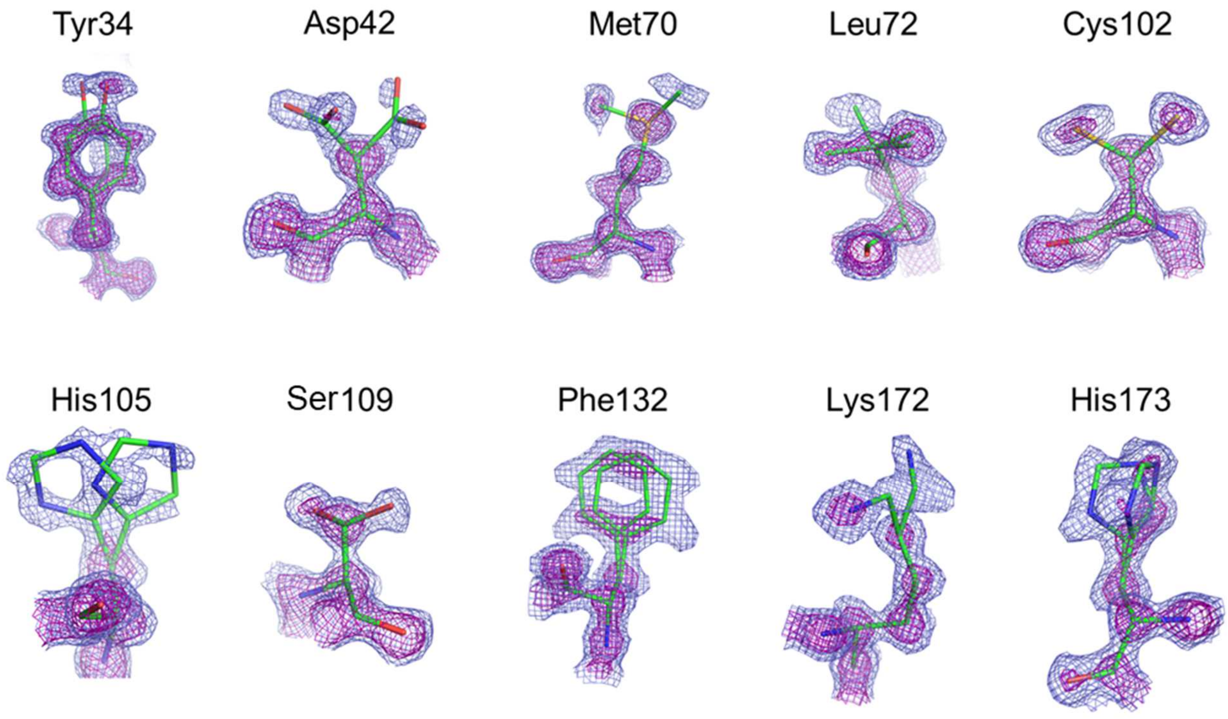

All the residues except for Tyr 34 are located on the protein surface exposed to solvent. The density levels of blue and purple nets are 2σ and 7σ, respectively.

Supplementary Fig. 6: Plots of peak distance for C-H, C-H₂, C-H₃, N-H, and O-H bonds.

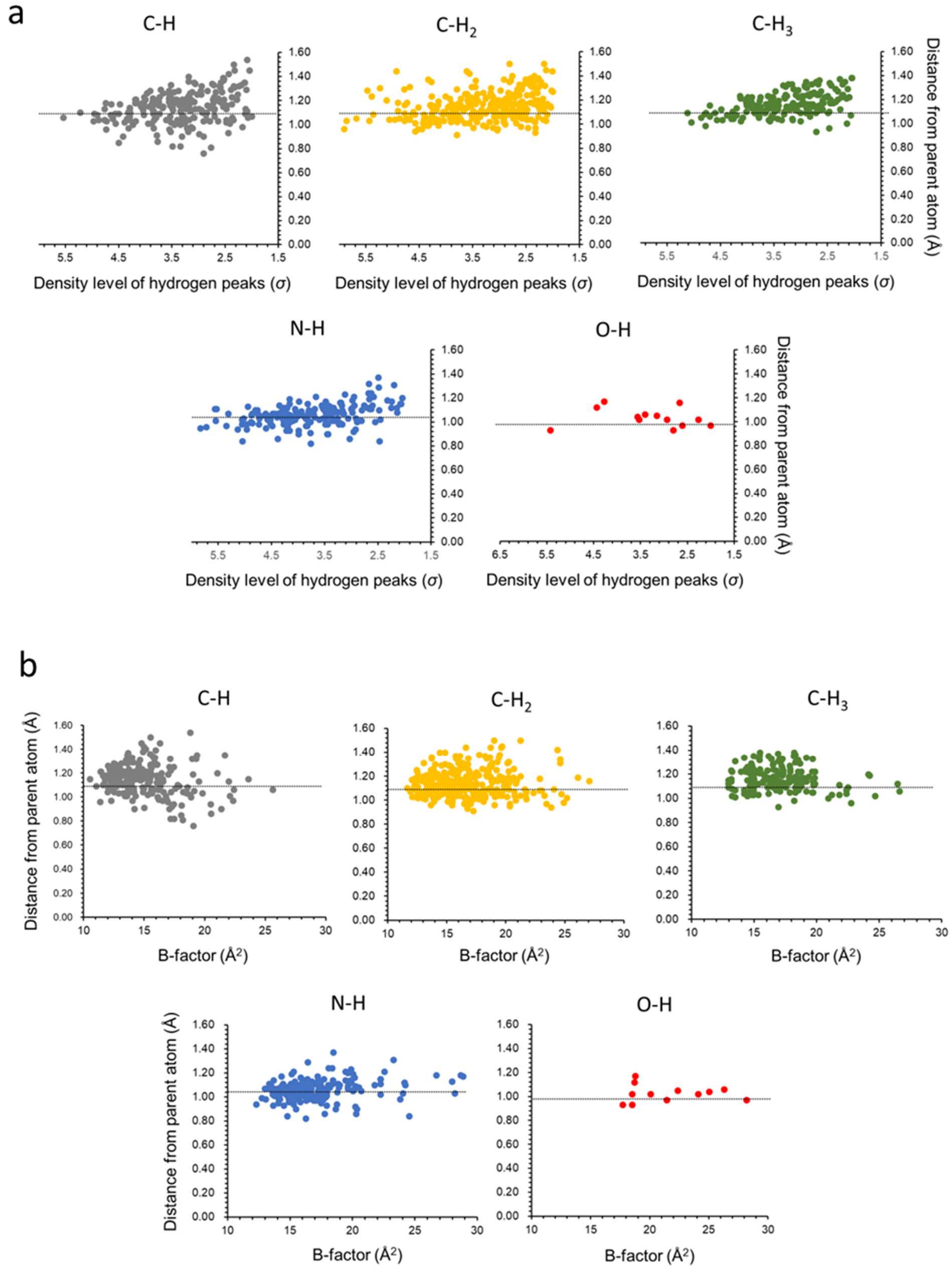

a Along display level (σ). **b** Along B-factor.

Supplementary Fig. 7: Plots of peak distance for C_{alk}-H and C_{aro}-H bonds.

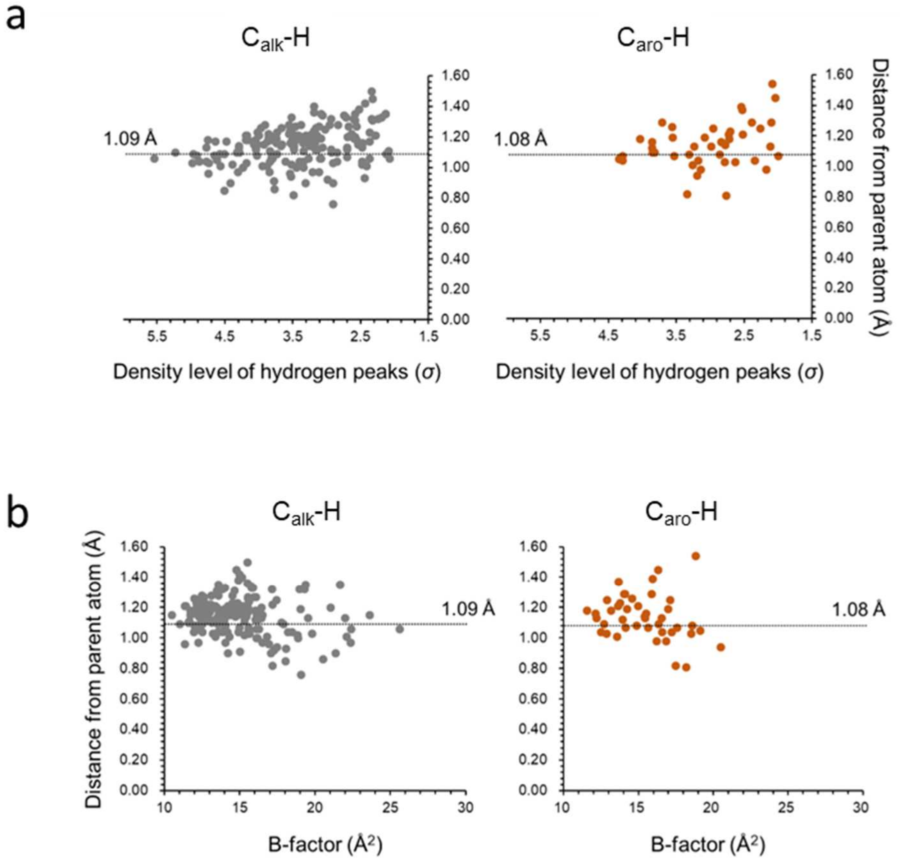

a Along display level (*σ*). **b** Along B-factor.

Supplementary Fig. 8: Plots of diffraction-component precision indices (DPI) for X-ray structures and $RMSD_{1/2}$ for single-particle reconstructions.

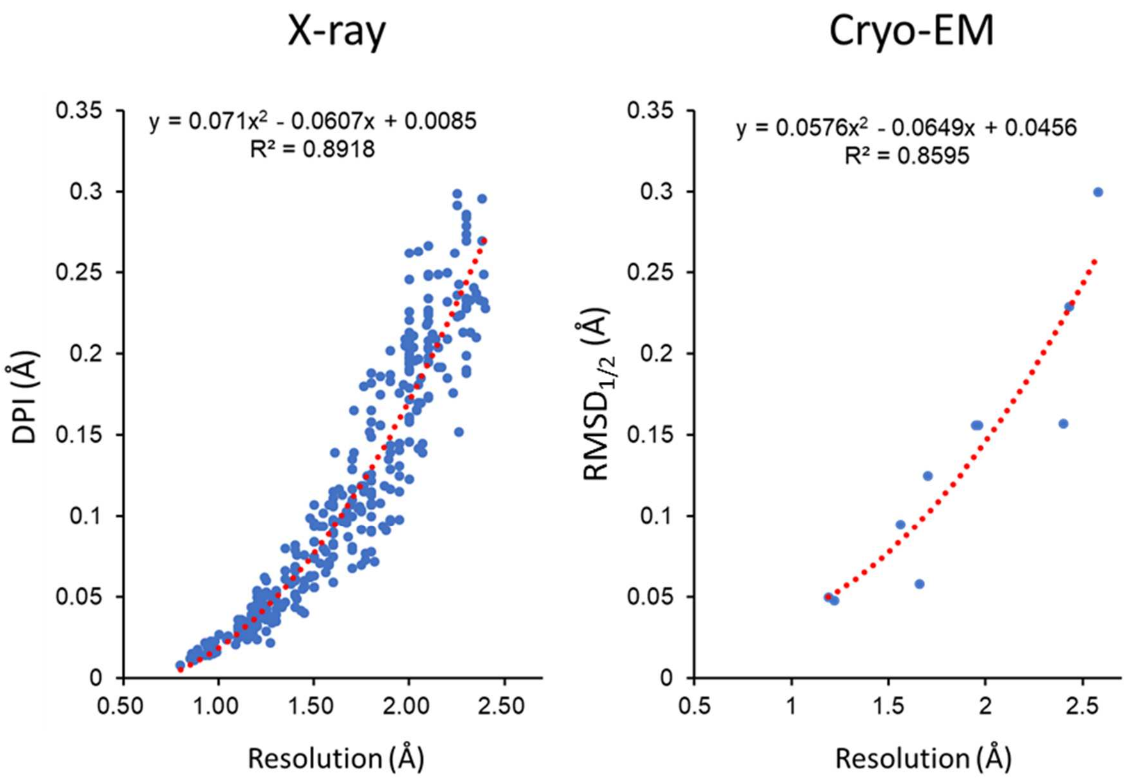

Fitted curves overlaid on the plots yield values in a similar range at a given resolution (e.g. DPI = 0.171 and $RMSD_{1/2}$ = 0.146 at 2.0 Å; 0.077 and 0.078 at 1.5 Å; 0.038 and 0.051 at 1.2 Å). DPI and $RMSD_{1/2}$ were calculated for 349 and for 10 structures, respectively. The number of tested cryo-EM structures is limited because there are many poorer-quality coordinates and no half maps deposited in the database. The PDB accession codes and values are given in Supplementary Table 2.

Supplementary References

1. T. Hamaguchi, S. Maki-Yonekura, H. Naitow, Y. Matsuura, T. Ishikawa, K. Yonekura, A new cryo-EM system for single particle analysis. *J. Struct. Biol.* **207**, 40–48 (2019).
2. H. Naitow, T. Hamaguchi, S. Maki-Yonekura, M. Isogai, N. Yoshikawa, K. Yonekura, Apple latent spherical virus structure with stable capsid frame supports quasi-stable protrusions expediting genome release. *Commun. Biol.* **3**, 488 (2020).
3. S. Maki-Yonekura, T. Hamaguchi, H. Naitow, K. Takaba, K. Yonekura, Advances in cryo-EM and ED with a cold-field emission beam and energy filtration —Refinements of the CRYO ARM 300 system in RIKEN SPring-8 center—. *Microscopy* **70**, 232–240 (2021).
4. K. Kato, N. Miyazaki, T. Hamaguchi, Y. Nakajima, F. Akita, K. Yonekura, J.-R. Shen, High-resolution cryo-EM structure of photosystem II: Effects of electron beam damage. *Commun. Biol.* **4**, 382 (2021).
5. T. Hamaguchi, K. Kawakami, K. Shinzawa-Itoh, N. Inoue-Kashino, S. Itoh, K. Ifuku, E. Yamashita, K. Maeda, K. Yonekura, Y. Kashino, Structure of the far-red light utilizing photosystem I of *Acaryochloris*, *Nat. Commun.* **12**, 2333 (2021).
6. J. Zivanov, T. Nakane, B. Forsberg, D. Kimanius, W. J. H. Hagen, E. Lindahl, S. H. W. Scheres, RELION-3: new tools for automated high-resolution cryo-EM structure determination. *eLife* **7**, e42166 (2018).
7. A. Punjani, J. L. Rubinstein, D. J. Fleet, M. A. Brubaker, cryoSPARC: algorithms for rapid unsupervised cryo-EM structure determination. *Nat. Methods* **14**, 290–296 (2017).
8. D. Tegunov, P. Cramer, Real-time cryo-electron microscopy data preprocessing with Warp. *Nat. Methods*. **16**, 1146-1152 (2019).
9. D. N. Mastronarde, Automated electron microscope tomography using robust prediction of specimen movements. *J. Struct. Biol.* **152**, 36–51 (2005).
10. S. Chen, G. McMullan, A. R. Faruqi, G. N. Murshudov, J. M. Short, S. H. Scheres, R. Henderson, High-resolution noise substitution to measure overfitting and validate resolution in 3D structure determination by single particle electron cryomicroscopy. *Ultramicroscopy* **135**, 24–35 (2013).
11. R. G. Efremov, A. Stroobants, Coma-corrected rapid single-particle cryo-EM data collection on the CRYO ARM 300. *Acta Cryst.* **D77**, 555–564 (2021).
12. R. M. Glaeser, D. Typke, P. C. Tiemeijer, J. Pulokas, A. Cheng, Precise beam-tilt alignment

and collimation are required to minimize the phase error associated with coma in high-resolution cryo-EM. *J. Struct. Biol.* **174**, 1-10 (2011).

13. A. Cheng, E. T. Eng, L. Alink, W. J. Rice, K. D. Jordan, L. Y. Kim, C. S. Potter, Bridget Carragher, High resolution single particle cryo-electron microscopy using beam-image shift. *J. Struct. Biol.* **204**, 270-275 (2018).
14. P. B. Rosenthal, R. Henderson, Optimal determination of particle orientation, absolute hand, and contrast loss in single-particle electron cryomicroscopy. *J. Mol. Biol.* **333**, 721–745 (2003).
15. K. M. Yip, N. Fischer, E. Paknia, A. Chari, H. Stark, Atomic-resolution protein structure determination by cryo-EM. *Nature* **587**, 157–161 (2020).
16. D. W. J. Cruickshank, Remarks about protein structure precision. *Acta Cryst.* **D55**, 583–601 (1999).
17. J. L. Chambers, R. M. Stroud, The accuracy of refined protein structures: comparison of two independently refined models of bovine trypsin. *Acta Cryst.* **B35**, 1861–1874 (1979).
18. R. A. Nicholls, M. Tykac, O. Kovalevskiy, G. N. Murshudov, Current approaches for the fitting and refinement of atomic models into cryo-EM maps using CCP-EM. *Acta Cryst.* **D74**, 492–505 (2018).
19. G. N. Murshudov, Refinement of atomic structures against cryo-EM maps. *Methods Enzymol.* **579**, 277-305 (2016).
20. R. A. Nicholls, M. Tykac, O. Kovalevskiy, G. N. Murshudov, Current approaches for the fitting and refinement of atomic models into cryo-EM maps using CCP-EM. *Acta Cryst.* **D74**, 492-505 (2018).
21. K. Yamashita, C. M. Palmer, T. Burnley, G. N. Murshudov, Cryo-EM single-particle structure refinement and map calculation using *Servalcat*. *Acta Cryst.* **D77**, 1282–1291 (2021).
22. G. Pintilie et al., Measurement of atom resolvability in cryo-EM maps with Qscores. *Nat. Methods* **17**, 328–334 (2020).
23. C. J. Williams et al., MolProbity: More and better reference data for improved all-atom structure validation. *Protein Sci.* **27**, 293–315 (2018).
